# Discovery, Design, Synthesis, and Application of Nucleoside/Nucleotides

**DOI:** 10.3390/molecules25071526

**Published:** 2020-03-27

**Authors:** Katherine Seley-Radtke

**Affiliations:** University of Maryland, Baltimore County, 1000 Hilltop Circle, Baltimore, MD 21250, USA; kseley@umbc.edu; Tel.: +1-410-455-8684; Fax: +1-410-455-2609

For decades, nucleosides and nucleotides have formed the cornerstone of antiviral, antiparasitic and anticancer therapeutics and have been used as tools in exploring nucleic acid structure and function. [1,2] This phenomenon is a direct result of their close structural similarity to naturally occurring nucleosides. As such, any changes to their diverse scaffolds can have profound effects. In general, nucleoside and nucleotide analogues target key biological pathways in the replication cycles of many diseases; however, some have also been shown to target human enzymes, which can sometimes result in harmful consequences. In that regard, this Special Issue will focus on some of the leading approaches towards design, synthesis and biological investigations, as well as the various applications for this highly relevant class of compounds. 

Two of the notable papers included in this Special Issue focused on novel nucleoside scaffolds, for example, Prof. Christian Ducho’s muraymycin nucleoside analogues. [3] His paper highlights successes in using these natural product nucleoside analogues for antibacterial therapies targeting the peptidoglycan synthetic pathway. Another highly unique nucleoside scaffold is the focus of one of the contributions from the Seley-Radtke group. The innovative fleximers have shown potent activity against two coronaviruses - SARS and MERS [4], as well as filoviruses such as Ebola, Sudan and Marburg. [5] Expanding on those results, a focused structure activity relationship study to improve upon the anti-Ebola activity previously noted is reported in this issue. [6]

Another well-known nucleoside scaffold, the carbocyclic analogues, were highlighted in a number of the papers in this Special Issue. For example, an excellent contribution from a highly multi-country and multidisciplinary team of investigators including Dr. Anastasia Khandazhinskaya, Robert Buckheit, Profs. Seley-Radtke and Harry de Koning, describes investigations into the antiprotozoan and antibacterial effects of some structurally unique carbocyclic nucleoside analogues. [7] Another paper from the Khandazhinskaya group focuses on some bicyclic pyrrolo- and furano [2,3-*d*]pyrimidine analogues. [8] A third describes the preparation of some novel cyclobutene nucleoside analogues from the El-Emam laboratories [9], while another from Slita [10] et al. looked at modifying the 1’-position of the carbocyclic scaffold. Their paper on homocarbocyclic nucleoside analogues also investigated a novel optically active sugar containing a bicyclo[2.2.1]heptane fragment.

Additional synthetic efforts to make modified nucleosides, in particular, the total synthesis of two well-known 5′-deoxy 7-deaza nucleoside analogues—toyocomycin and sangivamycin—were described in a paper by the Xiao group [11], while the contribution from the Sun group focused on modifying the sugar. Their paper describes the synthesis of a series of UDP-furanoses using a new synthetic route which employs uridine phosphoropiperidate. [12]

Aptamers were also a focus for this issue, starting with a computational investigation from Dr. Stacy Wetmore’s group, focused on studying the thrombin-DNA aptamer protein complex using molecular dynamics MD simulations. [13] Their simulations revealed that the chemically-modified base imparted noticeable structural changes to the aptamer without affecting the global conformation. Related to this, the paper from Yang et al. describes another use for dual targeting aptamers in cancer studies. These highly unique bispecific aptamers can potentially serve as a novel strategy for the targeted enhancement of antitumor immune reactions against MUC1-expressing malignancies. [14] 

Several other papers in this Special Issue described new RNA structural studies, including one from the Xu et al., which is focused on an investigation of nucleoside stabilizers for Z-form RNA. [15] Another excellent paper from Prof. Maria Camarasa’s group describes their efforts towards the design, synthesis and X-ray crystallographic studies of a series of compounds targeting the foot and mouth disease (FMDV) RNA-dependent RNA polymerase. [16] Their results may prove useful in the design of new antivirals against not only FMDV but also other picornaviruses, since all members of this family require uridylylation to initiate the viral RNA synthesis. 

Another structure study focused on solving issues by studying structure and function with NMR spectroscopy. Nucleic acid structure and dynamic studies by NMR spectroscopy suffer from chemical shift overlap and line broadening, both of which become worse as RNA size increases. In their paper, the Dayie group described the solid phase synthesis of a stable isotope labeled RNA to help elucidate the structure and function using NMR spectroscopy. [17] Finally, Zhou et al. published a report on using sequencing-based molecular markers and to study the Betula alnoides, a special type of birch tree found in many parts of Asia. [18]

In summary, an excellent series of papers covering a broad variety of topics were brought together in this Special Issue that focused on the design, synthesis and various applications for nucleoside, nucleotide and nucleic acid analogues. It is our hope that these will provide important information for others working on related projects, and provide the foundation and impetus for future studies. 
